# Impact of *Helicobacter pylori* Immunoglobulin G Levels and Atrophic Gastritis Status on Risk of Metabolic Syndrome

**DOI:** 10.1371/journal.pone.0166588

**Published:** 2016-11-16

**Authors:** Atsushi Takeoka, Jun Tayama, Hironori Yamasaki, Masakazu Kobayashi, Sayaka Ogawa, Tatsuo Saigo, Masaki Hayashida, Susumu Shirabe

**Affiliations:** 1 Unit of Preventive Medicine, Nagasaki University Graduate School of Biomedical Sciences, Nagasaki, Japan; 2 Graduate School of Education, Nagasaki University, Nagasaki, Japan; 3 Department of Endocrinology and Metabolism, Sasebo City General Hospital, Sasebo, Japan; 4 Center for Health and Community Medicine, Nagasaki University, Nagasaki, Japan; National Cancer Center, JAPAN

## Abstract

**Background:**

*Helicobacter pylori* (HP) infection is implicated in gastric and extra-gastric diseases. While gastritis-related chronic inflammation represents a known trigger of metabolic disturbances, whether metabolic syndrome (MetS) is affected by gastritis status remains unclear. We aimed to clarify the effect of HP-related gastritis on the risk of MetS.

**Materials and Methods:**

We retrospectively enrolled patients undergoing screening for MetS between 2014 and 2015. Investigations included HP-specific immunoglobulin G (IgG) antibody assays to detect HP infection, and serum pepsinogen assays to evaluate atrophic gastritis status. The risk of MetS was evaluated via multiple logistic regression analyses with two covariates: serum HP infection status (IgG levels) and atrophic gastritis status (two criteria were applied; pepsinogen I/II ratio < 3 or both pepsinogen I levels ≤ 70 μg/L and pepsinogen I/II ratio < 3).

**Results:**

Of 1,044 participants, 247 (23.7%) were HP seropositive, and 62 (6.0%) had MetS. HP seronegative and seropositive patients had similar risks of MetS. On the other hand, AG (defined in terms of serum PG I/II <3) was significant risk of MetS (OR of 2.52 [95% CI 1.05–7.52]). After stratification according to HP IgG concentration, patients with low HP infection status had the lowest MetS risk (defined as an odds ratio [OR] adjusted for age, sex, smoking, drinking and physical activity status). Taking this result as a reference, patients with negative, moderate, and high HP infection status had ORs (with 95% confidence intervals [CI]) of 2.15 (1.06–4.16), 3.69 (1.12–16.7), and 4.05 (1.05–26.8).

**Conclusions:**

HP-associated gastritis represents a risk factor for MetS. Research should determine why low and not negative HP infection status is associated with the lowest MetS risk.

## Introduction

*Helicobacter pylori* (HP) is a spiral-shaped bacterium that resides in the human gastric mucosal layer or adheres to the epithelial lining of the stomach. HP infection usually occurs in childhood and persists for a long time. HP infection is involved in gastric diseases such as chronic gastritis, gastric ulcer, and gastric adenocarcinoma [1, 2]. Although most HP infections are limited to the stomach, associations with certain extra-gastric manifestations has been noted, including iron-deficiency anemia [3], vitamin deficiency [4, 5], obesity [6], impaired glucose tolerance [7], insulin resistance [8], and cardiovascular diseases [9].

Metabolic syndrome (MetS) is an insulin-resistant state induced by a combination of risk factors that precedes-type 2 diabetes and may lead to increased cardiovascular morbidity. Recent evidence indicates that HP infection is also associated with MetS. Specifically, a cross-sectional study of a large population of Japanese adults evaluated the concentration of HP-specific immunoglobulin G (IgG) and found that HP-infected subjects had a significantly higher risk of MetS [10]. Another study reported a similar relationship [11], while others did not [12, 13]. These discrepancies may be related to the variation in HP-infection and gastritis status in the study populations. The underlying mechanisms of the MetS-HP relationship remain unknown, although chronic inflammation is presumed to play a key role. Specifically, HP infection induces chronic gastritis and local chronic inflammation, stimulating the production of response inflammatory proteins and cytokines such as C-reactive protein, tumor necrosis factor alpha (TNF-α), interleukins (IL-1, IL-6, IL-8, IL-10), and eicosanoids [14]. As overexpression of these proteins contributes to the pathogenesis of MetS [15], therefor the risk of MetS is it is expected to be affected by the inflammatory burden associated with HP infection.

From a different perspective, HP infection may also be associated with malnutrition, as growth retardation is suspected in HP-infected children [16], and weight gain has also been observed after HP eradication [17, 18]. Francois et al. reported HP eradication altered circulating meal-associated leptin and ghrelin levels and body mass index (BMI) [19]. The decrease in gastric secretory function may partially account for this HP-related malnutrition, as the levels of gastric hormones such pepsinogen (PG) I, gastrin, and ghrelin are known to decrease with the progression of atrophic gastritis (AG) [20]. The decrease in ghrelin secretion induces loss of appetite, which influences food intake and body weight. This phenomenon likely affects the relationship between MetS and HP infection, suggesting an inverse effect of HP infection on overnutrition and related MetS pathogenesis.

Furthermore, some reports suggested that AG, but not HP infection status, is associated with BMI [21, 22]. Because most of AG were induced by HP infection, the results mean the effect of HP infection on BMI occur by way of AG. Therefor it is also important to evaluate the effect of AG on MetS. To our knowledge, there are few reports that investigated the association between AG and MetS [23].

In the present study, we investigated the effect of HP-infection status (defined in terms of the concentration of HP-specific IgG) and AG status (defined in terms of serum PG levels) on the risk of MetS.

## Methods

### Study Population

This study enrolled employees of Nagasaki University with middle to high socioeconomic and educational status who underwent a comprehensive health check-up between 2014 and 2015. The analysis included only data regarding the participants who completed the health check-up investigations including anthropometric measurements and blood tests. Further subjects were excluded based on the following criteria: receiving medical treatment for hyperlipidemia or diabetes mellitus; history of antibiotic treatment against HP; inflammatory disease; severe liver or renal dysfunction; and neoplasm.

The study was conducted at the Center for Health and Community Medicine of Nagasaki University in Nagasaki, Japan. All patient data were anonymized before analysis. The study was approved by the ethical committee of Nagasaki University Graduate School of Biomedical Sciences (approval no. 16062492), and written informed consent was obtained from all participants.

### Data Collection

All participants completed questionnaires regarding smoking habits (habitually smoking ≥ 1 cigarette per day), physical activity (walking > 30 minutes per day), and alcohol consumption (habitually drinking ≥ 1 alcoholic drink per week). Anthropometric measurements including height, weight, and waist circumference were taken using standardized techniques and calibrated equipment. BMI was defined as weight divided by height squared (kg/m^2^). Blood pressure was measured serially with an electrical sphygmomanometer (DM-3000; Japan Precision Instruments Inc., Gunma, Japan), after each participant had rested for at least 5 minutes.

Blood samples were collected after the patients had fasted overnight. The following levels were measured: fasting plasma glucose, glycated hemoglobin (via assays certified by the National Glycohemoglobin Standardization Program), total cholesterol, high-density lipoprotein cholesterol (HDL-C), low-density lipoprotein cholesterol (LDL-C), triglycerides, and uric acid.

### Definition of MetS

MetS was diagnosed based on the revised National Cholesterol Education Program's Adult Treatment Panel III guidelines [24] upon fulfilling ≥ 3 of the following criteria: abdominal obesity (waist circumference ≥ 90cm and ≥ 80cm for Asian men and women, respectively); triglyceride levels ≥ 150mg/dL; HDL cholesterol levels ≤ 40mg/dL and 50mg/dL for men and women, respectively; systolic/diastolic blood pressure ≥ 130/85mmHg or receiving medical treatment; and fasting plasma glucose levels ≥ 100mg/dL.

### Measurement of HP-Specific IgG Concentration

HP infection was diagnosed based on the levels of HP-specific IgG measured using a commercially available enzyme-linked immunosorbent assay kit (E-Plate Eiken *H*. *pylori* antibody kit; Eiken Chemical Co., Ltd., Tokyo, Japan). For the Japanese population, the sensitivity and specificity of this kit were reported to be 95.2–100% and 76.2–80.0%, respectively [25, 26]. As previously reported [27] the subjects were stratified into HP-infection groups according to the concentration of HP-specific IgG as follows: HP seronegative (< 10 U⁄mL), low HP-specific IgG levels (10–30 U⁄mL), moderate HP-specific IgG levels (30–50 U⁄mL), or high HP-specific IgG levels (> 50 U⁄mL).

### Definition of AG

Serum PG (manifested as PG I and PG II) concentration and the PG I/II ratio were shown to reflect the functional and morphologic status of the gastric mucosa. AG is typically associated with a lower PG I/II ratio because the serum levels of PG I decrease, while those of PG II remain fairly constant. We therefore diagnosed AG based on the serum levels of PG I and II, which were measured using the Lumipulse Presto pepsinogen I and Lumipulse Presto pepsinogen II kits (Fujirebio Inc., Tokyo, Japan). Subjects with PG I levels of < 70 μg/L and a PG I/II ratio of < 3 were diagnosed with AG [28]. This criteria showed a sensitivity of 70.5% and specificity of 97.0% for AG compared with histology have been reported in Japan [29]. The criteria is optimized for gastric cancer screening [28]. Several other reports defined AG as PG I/II ratio of < 3 solely [30, 31] and this criteria showed 83.3% sensitivity and 87.1% specificity to moderate—severe AG diagnosed with endoscopy [32]. We applied these two criteria on analysis.

### Statistical Analysis

**Fig 1:**
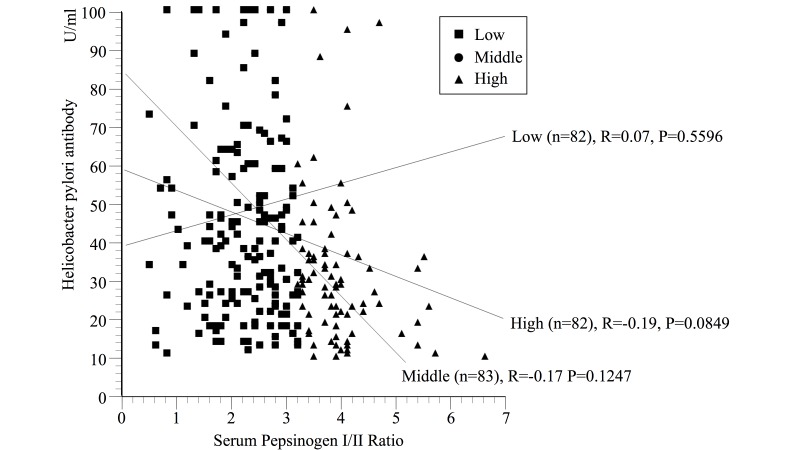
Correlation between Helicobacter pylori antibody and serum pepsinogen I/II ratio. Participants with low serum pepsinogen I/II ratio, middle serum pepsinogen I/II ratio, and high serum pepsinogen I/II ratio were plotted as ■, ● and ▲ respectively. Linear regression analysis showed a marginal negative correlation was observed between Helicobacter pylori antibody and serum pepsinogen I/II ratio in the low serum pepsinogen I/II ratio group (r = 0.19, p = 0.0849). There was no significant correlation between Helicobacter pylori antibody and serum pepsinogen I/II ratio in other groups.

All statistical analyses were performed using JMP version 10.0 (SAS Institute, Cary, North Carolina, USA). Student’s *t*-test and the chi-squared test were applied for continuous and categorical variables, respectively. The risk of MetS of HP infection and AG was assessed by logistic regression analysis. Two AG criteria were applied and both calculated separately. The risk of MetS, expressed in terms of sex and age adjusted odds ratios (ORs) with 95% confidence intervals (95% CIs), was calculated relative to the risk in the HP-seronegative group (Model 1, adjusted for sex and age). Sex and age adjusted ORs with 95% CIs of PG status (Model 2) and HP-seropositibity, PG status, smoking, drinking and physical activity status (Model 3) were also calculated. Thereafter, sex, age, smoking, drinking and physical activity status adjusted ORs with 95% CIs was calculated relative to the risk in the low HP-infection group (Model 4). In Model 5 we classified the participants into eight groups according to the combination of their HP infection status (defined in terms of IgG serum concentration) and AG status (defined in terms of serum PG levels), and compared the ORs of MetS within these eight groups. We also examined the interaction between HP IgG concentration and serum PG I/II ratio using linear regression model (Fig 1). The significance threshold was set at *p* < 0.05.

## Results

Of the 3,735 individuals who underwent the health check-up, 1,238 underwent anthropometric measurements and blood tests. After applying the exclusion criteria, a total of 1,044 participants (498 men) were enrolled in the study (Table 1). Of these, 247 (23.7%) were HP seropositive, and 62 (6.0%) were diagnosed with MetS. The HP-seropositive and seronegative groups differed in terms of age (46.6 ± 9.2 years and 42.9 ± 7.7 years, respectively; *p* < 0.0001) but not sex. Blood pressure was significantly higher in the HP-seropositive group (systolic blood pressure, 120.0 ± 16.0 versus 117.6 ± 14.1 mmHg, *p* = 0.03; diastolic blood pressure, 74.7 ± 12.1 versus 72.9 ± 11.1 mmHg, *p* = 0.003). Serum LDL-C concentration was significantly higher in the HP-seropositive group (120.3 ± 29.1 vs. 116.0 ± 30.4 mg/dL; *p* = 0.05), as were the serum levels of PG (*p* < 0.0001), although the PG I/II ratio was significantly lower in the HP-seropositive group, as expected (*p* < 0.0001). There were no differences between the HP-seropositive and seronegative groups in terms of smoking, drinking, and physical activity status.

**Table 1:** Characteristics of the study participants.

	All	HP-seropositive	HP-seronegative	*p-*value
Number of patients	1,044	247	797	
Age (years)	43.8 ± 8.2	46.6 ± 9.2	42.9 ± 7.7	< .0001
Male (%)	47.7	50.2	46.9	0.38
Height (cm)	164.6 ± 8.2	164.6 ± 8.1	164.5 ± 8.3	0.94
Weight (kg)	61.0 ± 12.7	61.7 ± 11.9	60.8 ± 12.9	0.32
Body mass index (kg/m^2^)	22.4 ± 3.5	22.7 ± 3.6	22.3 ± 3.5	0.10
Systolic blood pressure (mmHg)	118.2 ± 14.6	120.0 ± 16.0	117.6 ± 14.1	0.03
Diastolic blood pressure (mmHg)	73.3 ± 11.4	74.7 ± 12.1	72.9 ± 11.1	0.03
Triglyceride (mg/dL)	95.2 ± 70.8	93.4 ± 55.0	95.7 ± 75.1	0.66
High-density lipoprotein (mg/dL)	62.9 ± 14.2	61.8 ± 13.5	63.3 ± 14.5	0.16
Low-density lipoprotein (mg/dL)	117.0 ± 30.1	120.3 ± 29.1	116.0 ± 30.4	0.05
Fasting plasma glucose (mg/dL)	90.4 ± 12.4	89.9 ± 10.6	90.6 ± 12.9	0.40
Metabolic syndrome (n)	62	19	43	0.22
Pepsinogen I (ng/mL)	38.4 ± 14.6	48.6 ± 20.1	35.2 ± 10.7	< .0001
Pepsinogen II (ng/mL)	9.7 ± 7.0	18.5 ± 9.3	6.9 ± 2.3	< .0001
PG I/II	4.6 ± 1.4	2.9 ± 1.1	5.2 ± 1.0	< .0001
HP-specific IgG concentration (U/mL)	11.8 ± 19.4	39.9 ± 23.5	3.0 ± 0.4	< .0001
Low (U/mL)		103 (20.1 ± 5.6)		
Moderate (U/mL)		80 (39.0 ± 6.0)		
High (U/mL)		64 (72.9 ± 17.8)		
Smoking (%)	11.7	12.6	11.4	0.65
Drinking (%)	55	53.8	55.3	0.71
Physical activity (%)	21.3	24.3	20.3	0.18

HP: *Helicobacter pylori*; IgG: immunoglobulin G; PG: pepsinogen. Smoking was defined as habitually smoking more than one cigarette per day. Drinking was defined as habitually drinking more than one alcohol drink per week. Physical activity was determined by asking participants whether they walk more than 30 minutes per day.

The effect of HP-infection and AG on the risk of MetS was assessed by multiple logistic regression analyses (Table 2). No significant association was detected between positive HP-infection status and the risk of MetS after adjusting for age and sex (Model 1). On the other hand, AG (defined in terms of serum PG I/II <3) was significant risk of MetS (Model 2). When MetS risk of HP-infection status and AG status was calculated together, AG was significant risk but HP-infection lower the MetS risk (Model 3). The group with low HP infection (i.e., with lowest serum concentration of HP IgG) showed the lowest OR adjusted for age, sex, smoking, drinking, and physical activity status. Taking this group as a reference, patients with negative, moderate, and high HP infection status had ORs (with 95% confidence intervals [CI]) of 2.16 (1.06–4.16), 3.69 (1.12–16.7), and 4.05 (1.05–26.8). Smoking, drinking, and physical activity status did not have significant influence on the result (Model 3, 4).

**Table 2:** Odds ratios with 95% confidence intervals for *H*. *pylori* infection and pepsinogen status for metabolic syndrome.

	OR (95% CI)	*p*-value	OR (95% CI)	*p*-value
	*(A)*Atrophic gastritis is defined as PG I > 70 ng/mL and PG I/II < 3.0.		*(B)* Atrophic gastritis is defined as PG I/II < 3.0.	
Model 1: Age, Sex, and HP seropositivity				
HP seropositive/negative	0.85 (0.48–1.56)	0.59		
Model 2: Age, Sex, Atrophic gastritis				
Atrophic gastritis/Non-Atrophic gastritis	2.40 (0.94–8.16)	0.07	2.52 (1.05–7.52)	0.037
Model 3: Age, Sex, HP seropositivity, Atrophic gastritis, Smoking, Drinking and Physical activity				
HP seropositive/negative	0.52 (0.28–1.01)	0.054	0.42 (0.22–0.84)	0.016
Atrophic gastritis/Non-Atrophic gastritis	3.92 (1.34–14.3)	0.011	4.9 (1.75–16.0)	0.002
Model 4: Age, Sex, HP IgG level, Smoking, Drinking and Physical activity				
HP-seronegative	2.15 (1.06–4.16)	0.034		
Low HP infection (reference)	1	-		
Moderate HP infection	3.69 (1.12–16.7)	0.03		
High HP infection	4.05 (1.05–26.8)	0.042		
Model 5: Combination of HP IgG level and atrophic gastritis status				
Non atrophic gastritis				
HP-seronegative	3.44 (1.61–6.94)	0.002	3.55 (1.61–7.32)	0.003
Low HP infection (reference)	1	-	1	-
Moderate HP infection	5.22 (1.29–35.3)	0.018	3.47 (0.83–23.9)	0.093
High HP infection	5.99 (1.04–114.0)	0.045	3.70 (0.62–71.3)	0.17
Atrophic gastritis	¥			
Low HP infection	6.32 (1.54–43.1)	0.009	4.85 (1.34–23.3)	0.015
Moderate HP infection	7.30 (1.30–137.7)	0.021	11.3 (2.00–213)	0.004
High HP infection	8.54 (1.52–161.0)	0.011	11.5 (2.02–217)	0.003

HP: *Helicobacter pylori*; IgG: immunoglobulin G; PG: pepsinogen; CI: confidence interval; OR: odds ratio. In analysis including atrophic gastritis status (Model 2, 3, 5), atrophic gastritis was defined as: (A) PG I > 70 ng/mL and PG I/II < 3.0, (B) PG I/II < 3.0. Smoking was defined as habitually smoking more than one cigarette per day. Drinking was defined as habitually drinking more than one alcoholic drink per week. Physical activity was determined by asking participants whether they walk more than 30 minutes per day.

The participants were stratified according to the combination of HP infection and AG (Model 5). AG was not noted among HP-seronegative participants. Therefor analysis was performed within seven groups. The subgroup with low HP infection and no AG showed the lowest OR adjusted for age and sex. In examination of the interaction between HP IgG concentration and serum PG I/II ratio using linear regression model (Fig 1), a marginal negative correlation was observed between HP IgG and serum PG I/II ratio in the high serum PG I/II ratio group (r = 0.19, p = 0.0849). There was no significant correlation between HP IgG and serum PG I/II ratio in other groups.

## Discussion

In present study we observed differences of MetS risk according to AG and HP IgG concentrations. The findings were; 1) AG as defined PG I/II <3 is a significant risk of MetS. 2) Low H.pylori IgG concentration with non-AG group represented lower risk of MetS compared with H.pylori IgG negative, moderate and high concentration groups.

In most cases, HP infection occurs during childhood, but persists a long time and induces chronic gastritis. In the process of inflammation, HP-specific IgG is produced by activated immunocytes, and the concentration of HP-specific IgG reflects the progression of gastritis. Kishikawa *et al*. demonstrated that the HP-specific IgG titer was associated with the degree of gastritis progression in a positive and negative manner in individuals diagnosed with non-atrophic and AG, respectively [27].

As gastritis progresses, the time course of HP-specific IgG levels exhibits a unimodal shape. The early stage of HP-associated gastritis is indicated by low HP-specific IgG concentration with non-AG status, which, in our study, was associated with the lowest risk of MetS. Due to the low risk group, the OR of HP seropositive group were lowered (Table 2 model 3, 6). There may be several explanations for our finding that HP-seronegative individuals had higher risk of MetS than did those with low levels of HP-specific IgG. First, HP infection is known to affect micronutrient metabolism [3–5, 33], and several cross-sectional studies have indicated that HP infection causes growth retardation in children [34–36]. Observing 295 children over 3.7 years, after adjusting for other covariates, Mera *et al*. noted that children who were always HP-negative or who achieved successful HP clearance grew significantly faster than those who remained HP-positive [15]. Moreover, in adults, HP eradication has been shown to be followed by weight gain [19, 37]. In contrast, some reports have indicated that HP-related gastric symptoms, not HP infection itself, induce malnutrition [16, 38]. While the effect of this phenomenon on our conclusions cannot be quantified at the moment, our observation that low HP infection, and not negative HP infection status, is associated with the lowest risk of MetS might be explained by the inverse effect of HP infection and associated gastric symptoms on overnutrition. Moreover, several questions remain unanswered. For instance, do these HP-related malnutritional effects occur in early gastritis? Alternatively, if HP infection occurs in childhood, what are the reasons for phenotypical heterogeneity (different levels of HP-specific IgG and gastritis status) in adults? It is possible that certain participants had a specific phenotype for HP infection? Because the levels of HP-specific IgG reflect the inflammatory burden induced by HP infection [39], the participants who showed low HP-specific IgG concentration with non-AG status are considered to have some specific feature that can maintain the effect of HP infection minimally. Low MetS risk in this group indicate that they have common phenotype to prevent progression of both HP infection and MetS. It was previously reported that genetic polymorphisms in host alleles associated with pro-inflammatory and anti-inflammatory cytokines such as IL-1β, TNF-α, IL-6, and IL-10 affect the phenotype of HP-related diseases [40–43]. Polymorphism in these cytokines associated genes affect gastric inflammation and HP colonization. As these cytokines also play important roles in the pathogenesis of MetS [14], such polymorphisms may affect the prevalence of MetS. There is a possibility that we observed a population who have specific polymorphism which prevent progression of both HP infection and MetS. Furthermore, recent studies suggested the relationship of regulatory T cells (Treg cells) with MetS prevalence and HP infection. Specifically, reduced percentages of CD4(+)Foxp3(+) Treg cells were found in the abdominal fat of mice with genetic or diet-induced obesity [44]. In human studies, the percentages of Treg cells in the peripheral blood were significantly lower in children with MetS than those in healthy children [45], and similar results were noted in obese adults [46]. On the other hand, Treg cells were shown to attenuate HP infection and gastric inflammation [47, 48]. For example, an animal study reported that CD25+/Foxp3+ T cells regulate gastric inflammation including IgG production and HP colonization [47]. Therefore, individuals with high Treg cell function might be protected against MetS pathogenesis and HP-related gastritis. These phenotypical differences might be manifested in our study population and this may be the reason why the participants who showed low HP-specific IgG concentration with non-AG status have lower risk of MetS than HP IgG negative participants.

Our multiple logistic regression analysis revealed that AG was a significant risk factor for MetS. Furthermore, the addition of AG as a covariate weakened the significance of MetS risk in the moderate to high HP IgG concentration groups. This suggested that another background factor is at play between the two factors. The factor is most likely gastric inflammation. HP IgG concentration was reported to reflect serum IL-6 levels [49]. IL-6 is one of the important cytokines that mediate humoral immunity and plays a role in the pathogenesis of gastritis and MetS. In the present study, we observed a trend that MetS risk was increased according to HP IgG concentration. This finding supports the hypothesis that gastric chronic inflammation induces MetS [9–10].

In progressive AG, HP IgG is known to decrease and seronegativity is noted [50]. Therefore, AG with HP-seronegative status represents advanced-stage gastritis. Although we examined the interaction between HP IgG concentration and serum PG I/II ratio to clarify the effects of AG on the HP infection (Fig 1), we could not show the phenomenon that HP IgG concentration decrease along with PG I/II ratio lowering in progressive AG status clearly. In the present study, no participant had AG with HP-seronegative status and there were little subjects who have progressive AG. This characteristics of participants made it difficult to show the correlation between HP IgG and serum PG I/II in progressive AG. According to previous studies [51], in progressive AG, the amount of HP decrease and inflammation stabilizes. Gastric secretory function is disturbed and the secretion of gastric peptides like gastrin and ghrelin decreases [20]. Further research is needed how these changes effect on metabolic status.

This study has some limitations. First, this was a single-center, cross-sectional study. Therefore, we could not detect a causal relationship, and the generalization of our results should be considered with caution. The sample size used in the present study was smaller than that of the studies that showed significant MetS-HP relationships (< 3,000 participants), but larger than that of studies showing non-significant relationships (> 1000 participants) [52]. Discrepancies between the two criteria of AG and wide 95% CI might be caused by this small sample size. As described in method section, the AG criteria defined as PG I/II < 3 is reported to show relatively higher sensitivity than definition of AG as PG I < 70 μg/L and a PG I/II < 3, this different sensitivity may reflect the different results in evaluation of MetS risk for AG. In larger sample, the risk will be significant in both criteria. This study showed a weak trend of MetS risk in the HP seronegative group and moderate to high HP IgG concentration groups, with an OR of 1.84 and 95% CI of 0.77–5.50 (*p* = 0.18). However, in large populations, such differences are presumed to be clearly detectable. Second, we did not perform HP virulence tests, measurement of inflammation markers, and measurement of serum ghrelin levels in this study. The investigation of these factors will reveal the precise mechanism involved in the relationship between MetS and HP Further research is needed to reveal the precise mechanism of this phenomenon, and may yield important piece evidence that sheds light on the relationship between MetS and HP.

In conclusion, HP-associated gastritis appears to represent a risk factor for MetS. However, future research will need to determine why low and not negative HP infection status is associated with the lowest risk of MetS.

## Abbreviations

IgG: immunoglobulin G
HDL: high-density lipoprotein cholesterol (HDL-C)
HP: Helicobacter pylori
LDL-C: low-density lipoprotein cholesterol
MetS: metabolic syndrome
OR: odds ratio
PG: pepsinogen
AG: atrophic gastritis

## References

[1] Correa P, Piazuelo MB. Natural history of *Helicobacter pylori* infection. Dig Liver Dis 2008; 40: 490–496. 10.1016/j.dld.2008.02.035 18396115 PMC3142999

[2] Malfertheiner P, Megraud F, O'Morain CA, Atherton J, Axon ATR, Bazzoli F, et al Management of Helicobacter pylori infection—The Maastricht IV/ Florence consensus report. Gut 2012;61(5):646–664. 10.1136/gutjnl-2012-302084 22491499

[3] Muhsen K, Cohen D. *Helicobacter pylori* infection and iron stores: A systematic review and meta-analysis. Helicobacter 2008; 13: 323–340. 10.1111/j.1523-5378.2008.00617.x 19250507

[4] Kaptan K, Beyan C, Ural AU, Çetin T, Avcu F, Gülşen M, et al Helicobacter pylori—Is it a novel causative agent in vitamin B12 deficiency? Arch Intern Med 2000;160(9):1349–1353. 10809040 10.1001/archinte.160.9.1349

[5] Sobala GM, Schorah CJ, Shires S, Lynch DAF, Gallacher B, Dixon MF, et al Effect of eradication of Helicobacter pylori on gastric juice ascorbic acid concentrations. Gut 1993;34(8):1038–1041. 8174949 10.1136/gut.34.8.1038 PMC1374349

[6] Arslan E, Atilgan H, Yavaşoǧlu I. The prevalence of *Helicobacter pylori* in obese subjects. Eur J Intern Med 2009; 20: 695–697. 10.1016/j.ejim.2009.07.013 19818289

[7] Chen Y, Blaser MJ. Association between gastric helicobacter pylori colonization and glycated hemoglobin levels. J Infect Dis 2012;205(8):1195–1202. 10.1093/infdis/jis106 22427676 PMC3308905

[8] Polyzos SA, Kountouras J, Zavos C, Deretzi G. The Association Between Helicobacter pylori Infection and Insulin Resistance: A Systematic Review. Helicobacter 2011;16(2):79–88. 10.1111/j.1523-5378.2011.00822.x 21435084

[9] Nam SY, Ryu KH, Park BJ, Park S. Effects of Helicobacter pylori Infection and Its Eradication on Lipid Profiles and Cardiovascular Diseases. Helicobacter 2015;20(2):125–132. 10.1111/hel.12182 25382033

[10] Gunji T, Matsuhashi N, Sato H, Fujibayashi K, Okumura M, Sasabe N, et al Helicobacter pylori infection is significantly associated with metabolic syndrome in the Japanese population. Am J Gastroenterol 2008;103(12):3005–3010. 10.1111/j.1572-0241.2008.02151.x 19086952

[11] Chen TP, Hung HF, Chen MK, Lai HH, Hsu WF, Huang KC, et al Helicobacter Pylori Infection is Positively Associated with Metabolic Syndrome in Taiwanese Adults: A Cross-Sectional Study. Helicobacter 2015;20(3):184–191. 10.1111/hel.12190 25582223

[12] Naja F, Nasreddine L, Hwalla N, Moghames P, Shoaib H, Fatfat M, et al Association of H. pylori Infection with Insulin Resistance and Metabolic Syndrome Among Lebanese Adults. Helicobacter 2012;17(6):444–451. 10.1111/j.1523-5378.2012.00970.x 23066847

[13] Chen LW, Chien CY, Yang KJ, Kuo SF, Chen CH, Chien RN. Helicobacter pylori Infection increases insulin resistance and metabolic syndrome in residents younger than 50 years old: A community-based study. PLoS ONE 2015;10(5). 10.1371/journal.pone.0128671 PMC4447445 26020514

[14] D'Elios MM, Czinn SJ. Immunity, inflammation, and vaccines for *Helicobacter pylori*. Helicobacter 2014; 19: 19–26. 10.1111/hel.12156 25167941

[15] Eckel RH, Grundy SM, Zimmet PZ. The metabolic syndrome. Lancet 2005; 365: 1415–1428. 10.1016/S0140-6736(05)66378-7 15836891

[16] Mera RM, Bravo LE, Goodman KJ, Yepez MC, Correa P. Long-term effects of clearing Helicobacter pylori on growth in school-age children. Pediatr Infect Dis J 2012;31(3):263–266. 10.1097/INF.0b013e3182443fec 22315005 PMC3415984

[17] Lane JA, Murray LJ, Harvey IM, Donovan JL, Nair P, Harvey RF. Randomised clinical trial: Helicobacter pylori eradication is associated with a significantly increased body mass index in a placebo-controlled study. Aliment Pharmacol Ther 2011;33(8):922–929. 10.1111/j.1365-2036.2011.04610.x 21366634

[18] Furuta T, Shirai N, Xiao F, Takashima M, Hanai H. Effect of helicobacter pylori infection and its eradication on nutrition. Aliment Pharmacol Ther 2002;16(4):799–806. 11929399 10.1046/j.1365-2036.2002.01222.x

[19] Francois F, Roper J, Joseph N, Pei Z, Chhada A, Shak JR, et al The effect of H. pylori eradication on meal-associated changes in plasma ghrelin and leptin. BMC Gastroenterol 2011;11. 10.1186/1471-230X-11-37 PMC3089783 21489301

[20] Isomoto H, Ueno H, Saenko VA, Mondal MS, Nishi Y, Kawano N, et al Impact of Helicobacter pylori infection on gastric and plasma ghrelin dynamics in humans. Am J Gastroenterol 2005;100(8):1711–1720. 10.1111/j.1572-0241.2005.41492.x 16086706

[21] Watabe H, Mitsushima T, Derakhshan MH, Yamaji Y, Okamoto M, Kawabe T, et al Study of association between atrophic gastritis and body mass index: A cross-sectional study in 10,197 Japanese subjects. Dig Dis Sci 2009;54(5):988–995. 10.1007/s10620-008-0468-7 18787953

[22] Torisu T, Matsumoto T, Takata Y, Ansai T, Soh I, Awano S, et al Atrophic gastritis, but not antibody to Helicobacter pylori, is associated with body mass index in a Japanese population. J Gastroenterol 2008;43(10):762–766. 10.1007/s00535-008-2219-0 18958544

[23] Longo-Mbenza B, Nkondi Nsenga J, Vangu Ngoma D. Prevention of the metabolic syndrome insulin resistance and the atherosclerotic diseases in Africans infected by Helicobacter pylori infection and treated by antibiotics. Int J Cardiol 2007;121(3):229–238. 10.1016/j.ijcard.2006.12.003 17368586

[24] Alberti KGMM, Eckel RH, Grundy SM, Zimmet PZ, Cleeman JI, Donato KA, et al Harmonizing the metabolic syndrome: A joint interim statement of the international diabetes federation task force on epidemiology and prevention; National heart, lung, and blood institute; American heart association; World heart federation; International atherosclerosis society; And international association for the study of obesity. Circulation 2009;120(16):1640–1645. 10.1161/CIRCULATIONAHA.109.192644 19805654

[25] Matsuo K, Hamajima N, Suzuki T, Nakamura T, Matsuura A, Tominaga S. Better ROC curves for a regionally developed helicobacter pylori antibody test. Asian Pac J Cancer Preven 2001;2(2):155–156. 12718648

[26] Fujioka T, Tokieda M. [Validity of serum anti-*Helicobacter pylori* antibody using enzyme immunoassay for the diagnosis in eradication of *Helicobacter pylori*]. Jpn J Med Pharm Sci 2000; 43: 573–579. [Article in Japanese].

[27] Kishikawa H, Nishida J, Ichikawa H, Kaida S, Takarabe S, Matsukubo T, et al Fasting gastric pH of Japanese subjects stratified by IgG concentration against Helicobacter pylori and pepsinogen status. Helicobacter 2011;16(6):427–433. 10.1111/j.1523-5378.2011.00868.x 22059393

[28] Miki K. Gastric cancer screening using the serum pepsinogen test method. Gastric Cancer 2006; 9: 245–253. 10.1007/s10120-006-0397-0 17235625

[29] Watanabe Y, Kurata JH, Mizuno S, Mukai M, Inokuchi H, Miki K, et al Helicobacter pylori infection and gastric cancer: A nested case-control study in a rural area of Japan. Dig Dis Sci 1997;42(7):1383–1387. 9246033 10.1023/a:1018833819860

[30] Anderson LA, Murphy SJ, Johnston BT, Watson RGP, Ferguson HR, Bamford KB, et al Relationship between Helicobacter pylori infection and gastric atrophy and the stages of the oesophageal inflammation, metaplasia, adenocarcinoma sequence: Results from the FINBAR case-control study. Gut 2008;57(6):734–739. 10.1136/gut.2007.132662 18025067

[31] Kang JM, Kim N, Yoo JY, Park YS, Lee DH, Kim HY, et al The role of serum pepsinogen and gastrin test for the detection of gastric cancer in Korea. Helicobacter 2008;13(2):146–156. 10.1111/j.1523-5378.2008.00592.x 18321304

[32] Leja M, Kupcinskas L, Funka K, Sudraba A, Jonaitis L, Ivanauskas A, et al The Validity of a Biomarker Method for Indirect Detection of Gastric Mucosal Atrophy Versus Standard Histopathology. Dig Dis Sci 2009;54(11):2377–2384. 10.1007/s10620-009-0947-5 19731026

[33] Lahner E, Persechino S, Annibale B. Micronutrients (other than iron) and *Helicobacter pylori* infection: a systematic review. Helicobacter 2012; 17: 1–15. 10.1111/j.1523-5378.2011.00892.x 22221610

[34] Patel P, Mendall MA, Khulusi S, Northfield TC, Strachan DP. Helicobacter pylori infection in childhood: Risk factors and effect on growth. BMJ 1994;309(6962):1119 7987103 10.1136/bmj.309.6962.1119 PMC2541954

[35] Perri F, Pastore M, Leandro G, Clemente R, Ghoos Y, Peeters M, et al Helicobacter pylori infection and growth delay in older children. Arch Dis Child 1997;77(1):46–49. 9279151 10.1136/adc.77.1.46 PMC1717226

[36] Richter T, List S, Müller DM, Deutscher J, Uhlig HH, Krumbiegel P, et al Five- to 7-year-old children with Helicobacter pylori infection are smaller than Helicobacter-negative children: A cross-sectional population-based study of 3,315 children. J Pediatr Gastroenterol Nutr 2001;33(4):472–475. 11698766 10.1097/00005176-200110000-00010

[37] Azuma T, Suto H, Ito Y, Muramatsu A, Ohtani M, Dojo M, et al Eradication of Helicobacter pylori infection induces an increase in body mass index. Aliment Pharmacol Ther Suppl 2002;16(2):240–244. 10.1046/j.1365-2036.16.s2.31.x 11966548

[38] Gulcan M, Ozen A, Karatepe HO, Gulcu D, Vitrinel A. Impact of H. pylori on growth: Is the infection or mucosal disease related to growth impairment? Dig Dis Sci 2010;55(10):2878–2886. 10.1007/s10620-009-1091-y 20112067

[39] Tu H, Sun L, Dong X, Gong Y, Xu Q, Jing J, et al Serum anti-Helicobacter pylori immunoglobulin G titer correlates with grade of histological gastritis, mucosal bacterial density, and levels of serum biomarkers. Scand J Gastroenterol 2014;49(3):259–266. 10.3109/00365521.2013.869352 24329006

[40] El-Omar EM, Carrington M, Chow W-, McColl KEL, Bream JH, Young HA, et al Interleukin-1 polymorphisms associated with increased risk of gastric cancer. Nature 2000;404(6776):398–402. 10.1038/35006081 10746728

[41] Machado JC, Figueiredo C, Canedo P, Pharoah P, Carvalho R, Nabais S, et al A proinflammatory genetic profile increases the risk for chronic atrophic gastritis and gastric carcinoma. Gastroenterology 2003;125(2):364–371. 12891537 10.1016/s0016-5085(03)00899-0

[42] Lu W, Pan K, Zhang L, Lin D, Miao X, You W. Genetic polymorphisms of interleukin (IL)-1B, 1L-1RN, IL-8, IL-10 and tumor necrosis factor α and risk of gastric cancer in a Chinese population. Carcinogenesis 2005;26(3):631–636. 10.1093/carcin/bgh349 15579481

[43] Jung MK, Kim N, Dong HL, Ji HP, Mi KL, Joo SK, et al The effects of genetic polymorphisms of IL-6, IL-8, and IL-10 on Helicobacter pylori-induced gastroduodenal diseases in Korea. J Clin Gastroenterol 2009;43(5):420–428. 10.1097/MCG.0b013e318178d1d3 19077731

[44] Feuerer M, Herrero L, Cipolletta D, Naaz A, Wong J, Nayer A, et al Lean, but not obese, fat is enriched for a unique population of regulatory T cells that affect metabolic parameters. Nat Med 2009;15(8):930–939. 10.1038/nm.2002 19633656 PMC3115752

[45] Łuczyński W, Wawrusiewicz-Kurylonek N, Iłendo E, Bossowski A, Głowińska-Olszewska B, Kretowski A, et al Generation of functional T-regulatory cells in children with metabolic syndrome. Arch Immunol Ther Exp 2012;60(6):487–495. 10.1007/s00005-012-0198-6 23052042

[46] Wagner NM, Brandhorst G, Czepluch F, Lankeit M, Eberle C, Herzberg S, et al Circulating regulatory T cells are reduced in obesity and may identify subjects at increased metabolic and cardiovascular risk. Obesity 2013;21(3):461–468. 10.1002/oby.20087 23592653

[47] Rad R, Brenner L, Bauer S, Schwendy S, Layland L, da Costa CP, et al CD25+/Foxp3+ T Cells Regulate Gastric Inflammation and Helicobacter pylori Colonization In Vivo. Gastroenterology 2006;131(2):525–537. 10.1053/j.gastro.2006.05.001 16890606

[48] Harris PR, Wright SW, Serrano C, Riera F, Duarte I, Torres J, et al Helicobacter pylori Gastritis in Children Is Associated With a Regulatory T-Cell Response. Gastroenterology 2008;134(2):491–499. 10.1053/j.gastro.2007.11.006 18242215

[49] Nakagawa H, Tamura T, Mitsuda Y, Goto Y, Kamiya Y, Kondo T, et al Significant association between serum interleukin-6 and helicobacter pylori antibody levels among h. pylori -positive japanese adults. Mediators Inflamm 2013;2013. 10.1155/2013/142358 PMC3881527 24453409

[50] Plummer M, Vivas J, Fauchere JL, Del Giudice G, Pena AS, Ponzetto A, et al Helicobacter pylori and stomach cancer: A case-control study in Venezuela. Cancer Epidemiol Biomarkers Prev 2000;9(9):961–965. 11008915

[51] Kishikawa H, Kimura K, Takarabe S, Kaida S, Nishida J. Helicobacter pylori antibody titer and gastric cancer screening. Dis Markers 2015;2015. 10.1155/2015/156719 PMC4606161 26494936

[52] Upala S, Jaruvongvanich V, Riangwiwat T, Jaruvongvanich S, Sanguankeo A. Association between Helicobacter pylori infection and metabolic syndrome: a systematic review and meta-analysis. J Dig Dis 2016;17(7):433–440. 10.1111/1751-2980.12367 27273478

